# Neuroinflammation in inflammatory bowel disease

**DOI:** 10.1186/1742-2094-7-37

**Published:** 2010-07-08

**Authors:** Shaheen E Lakhan, Annette Kirchgessner

**Affiliations:** 1 Global Neuroscience Initiative Foundation, Los Angeles, CA, USA

## Abstract

Inflammatory bowel disease is a chronic intestinal inflammatory condition, the pathology of which is incompletely understood. Gut inflammation causes significant changes in neurally controlled gut functions including cramping, abdominal pain, fecal urgency, and explosive diarrhea. These symptoms are caused, at least in part, by prolonged hyperexcitability of enteric neurons that can occur following the resolution of colitis. Mast, enterochromaffin and other immune cells are increased in the colonic mucosa in inflammatory bowel disease and signal the presence of inflammation to the enteric nervous system. Inflammatory mediators include 5-hydroxytryptamine and cytokines, as well as reactive oxygen species and the production of oxidative stress. This review will discuss the effects of inflammation on enteric neural activity and potential therapeutic strategies that target neuroinflammation in the enteric nervous system.

## Introduction

Inflammatory bowel disease (IBD), which is comprised of two main types, ulcerative colitis (UC) and Crohn's disease (CD), affects approximately 3.6 million people in the United States and Europe. An alarming rise in previous low-incidence areas, such as Asia, is currently being observed [1]. Although considerable progress has been made in recent years, a major gap in knowledge of the pathogenesis of IBD remains. Without further research on the pathogenesis of IBD, the discovery of lasting, effective forms of treatment is impossible. Moreover, predicting disease outcome remains a challenge.

IBD is characterized by chronic or relapsing immune activation and inflammation within the gastrointestinal (GI) tract that markedly alters GI function [2]. In CD all layers of the gut may be involved, and normal healthy gut may be found between sections of diseased bowel. In contrast, UC causes inflammation and ulcers in the top layer lining the large intestine. When the gut is inflamed, there is breakdown of intestinal barrier function, abnormal secretion, changes in the patterns of motility, and visceral sensation, which contributes to symptom generation. Typically, alterations in gut function that accompany GI inflammation give rise to diarrhea, cramping, and pain, all standard IBD symptoms.

Other chronic inflammatory diseases of the gut, including celiac disease [3], an autoimmune reaction to gluten found in wheat and other grains, and irritable bowel syndrome (IBS) [4], are characterized by abdominal pain and GI dysfunction. There is clinical overlap between IBD and IBS, with IBS-like symptoms frequently reported in patients before the diagnosis of IBD, and a higher than expected percentage reports of IBS symptoms in patients in remission from established IBD [5]. There is growing evidence that occult inflammation in the GI mucosa, rather than coexistent IBS may play an important role in IBS-like symptoms in patients with IBD who are thought to be in clinical remission [5].

Inflammation is well known to affect gut function. Experimental data suggest that inflammation, even if mild, could lead to persistent changes in GI nerve and smooth muscle function, resulting in colonic dysmotility, hypersensitivity, and dysfunction even when the preceding infection is restricted to the proximal small intestine. Furthermore, alterations in gut function are observed after resolution of an acute intestinal inflammation, suggesting that inflammation-induced changes persist following recovery and play a major role in the generation of symptoms associated with IBD [6,7].

Data obtained from biopsies from patients with IBD and animal models of IBD have consistently suggested a role of inflammatory effects on enteric neurons in the generation of symptoms associated with IBD [8,9]. The enteric nervous system (ENS), the intrinsic innervation of the bowel, controls virtually all GI functions (e.g., motility, secretion, blood flow, mucosal growth and aspects of the local immune system). It is presently unknown whether the persistent alterations in gut function observed following inflammation are due to altered properties of enteric nerves.

This review will provide a brief overview of the current understanding of enteric neural abnormalities evoked by gut inflammation, particularly in IBD. Despite advances in the understanding of the pathophysiology of IBD, therapeutic options for combating functional changes that persist following transient GI inflammation are not available. Neuroprotective agents that can curtail the effects of inflammation on GI nerves may show potential in the treatment of chronic inflammatory diseases of the bowel.

## The enteric nervous system

The ENS is a component of the autonomic nervous system with the unique ability to function independently of the central nervous system (CNS) (see [10] for a review). The ENS regulates and coordinates almost all aspects of intestinal function including gut motility, the transport of fluid and electrolytes, the secretion of mucins, the production of cytokines, and the regulation of epithelial barrier function. Each of the aspects of physiology are compromised in IBD and it is therefore not surprising that there is an increasing amount of research interest in elucidating the role of the ENS in the pathogenesis of IBD.

Enteric ganglia are organized into two major ganglionated plexuses, the myenteric (Auerbach's) and submucosal (Meissner's) plexus. They contain a variety of functionally distinct neurons, including primary afferent neurons, interneurons, and motor neurons, synaptically linked to each other in microcircuits. In addition, enteric neurons are supported by glial cells, the ENS counterparts of astrocytes of the CNS, that can modulate enteric neuron function. Neurons of the ENS also exist in close apposition to cells of the mucosal immune system and the intestinal epithelium and bi-directional communication is known to occur at both of these interfaces.

Enteric neurons are known to control virtually all GI functions, including motility, secretion, blood flow, mucosal growth and aspects of the local immune system. Consequently, permanent or even transient structural alterations in the ENS, as occur in IBD, disrupt normal GI function.

## Effects of inflammation on enteric neurons

Patients with IBD manifest symptoms suggestive of disturbed gut function, such as sensory-motor changes and altered secretion. These abnormalities illustrate the impact of inflammatory signals generated within the gut mucosa on neural signaling in the ENS.

Inflammation-related alterations in the ENS can be divided into those that occur in the structural morphology of the ENS and those that occur in the levels of enteric neurotransmitters. Several studies have demonstrated ENS structural changes in human IBD (for review, see [9,11]). Tissue analysis of patients with CD or UC showed the existence of ENS abnormalities including ganglia and nerve bundles of increased size (hypertrophy) and/or number (hyperplasia) [9,12-14], as well as changes to glial cells (hyperplasia), including increased expression of the major histocompatability complex class II antigens [9].

Although changes in the morphology of the ENS are observed in both CD and UC, structural changes are more marked in CD than they are in UC [13]. Nerve trunk hypertrophy and hyperplasia have been reported mainly for the mucosa, submucosa and myenteric plexus of the ileum and colon of patients with CD, and these structural abnormalities are associated with the extent of inflammatory infiltrate. Mucosal and submucosal abnormalities are less commonly observed in patients with UC. In fact, based on electron microscopic examination, it has been suggested that severe and extensive necrosis of gut axons may differentiate CD from other inflammatory disorders [14]. Recurrence of CD after ileal or colonic resection is common.

The natural history of IBD progressively leads to the development of complications in approximately two-thirds of CD patients and less than one-third of UC patients [15]. In CD, the main complications are the development of fibrotic strictures that lead to intestinal obstruction and the development of intra-abdominal and peri-anal fistulae and abscesses. Recently, the presence of myenteric and submucosal plexitis were shown to be predictive of early endoscopic CD recurrence [16,17]. Thus, structural changes in the ENS are predictive of disease evolution suggesting that neuroprotection would decrease disease severity and may play a role in recurrence in CD.

Ganglion cell and nerve process degeneration and necrosis often accompany ENS structural changes in IBD. These findings have been confirmed in tissues from both CD and UC patients showing swollen, empty axons, filled with large membrane-bound vacuoles, swollen mitochondria, and concentrated neurofibrils [18]. Interestingly, these abnormalities in gut structure may also be observed in affected and non-affected areas of CD.

Structural ENS abnormalities -- including axonal hypertrophy and neuronal cell death -- have been observed in a variety of experiment animal models of colitis [19]. Animal models have been developed to better understand the pathophysiology of colitis, and are frequently used to evaluate new anti-inflammatory strategies. However, acute animal models of colitis do not exactly mimic human IBD since acute inflammation is a nonspecific pathological process in IBD. Nevertheless, there is a close relationship between inflammation and ENS structural changes and animal models of colitis, which can be used to elucidate the mechanisms underlying ENS dysfunction, whose understanding would be rather difficult in human diseases.

Experimental models of colitis have reported either no change in cell numbers or a significant loss of enteric neurons [20]. For example, Linden et al. reported that the number of myenteric neurons per ganglion are significantly decreased in trinitrobenzene sulfonic acid (TNBS)-induced colitis in guinea pigs [21]. TNBS-induced colitis results in a Th1-dominant inflammation with excessive production of IFN-γ, TNF-α, and IL-12. This model presumably resembles CD because of the mucosal inflammation mediated by a Th1 response. TNBS colitis is generally induced by intrarectal application of TNBS in ethanol [22].

TNBS colitis is associated with a 20% loss of myenteric neurons, which occurs at the time neutrophils infiltrate the ganglia. Interestingly, this neuronal loss persisted at a 56-day time-point, when inflammation had resolved [21]. The decrease in myenteric neurons was not associated with a decrease in any particular subpopulation of neurons, suggesting an indiscriminant loss of neurons that occurs during the onset of TNBS-colitis. In addition, the neurotoxic insult was followed by rapid regeneration of axons from the surviving neurons. Inflammation was also associated with a significant loss of myenteric neurons in rats with TNBS-induced colitis [23].

Similar findings were reported in 2,4-dinitrobenzenesulfonic acid (DNBS)-induced colitis in rats, where there was a 50% decrease of neuronal cells in the myenteric plexus [24]. Histopathological observation has shown that DNBS-induced damages resemble human UC [25]. Cell death was observed as early as 48 hours after the induction of colitis; a reduction which persisted for the 35 days of the study [24].

Significant neuronal death was also observed in *Trichinella spiralis *murine colitis and *Trichinella spiralis*-induced colitis in rats [26,27]. Intracolonic administration of *Trichinella spiralis *larvae in rats causes colitis with features similar to UC, notably with inflammation predominantly limited to the colonic mucosa [26]. Interestingly, in this animal model of colitis, there was a significant loss of nitric oxide synthase- (NOS-) immunoreactive neurons in the myenteric plexus of infected rats. Moreover, the selective loss of NOS-positive neurons appears to underlie changes in motility.

Why there are such differences in findings regarding alterations in numbers of nerve cells in human patients and animal models of IBD is uncertain, but it may be due to the duration of time over which the inflammation develops, with the rapid onset of inflammation in models employing chemicals such as TNBS and DNBS [28].

The mechanism of induction of neuronal cell death remains unknown; however, the loss of neurons was associated with the appearance of eosinophilic and neutrophilic infiltrates into myenteric ganglia suggesting that it may be mediated by interactions with the mucosal immune system [24].

Myenteric ganglionitis, associated with infiltrates of lymphocytes such as plasma cells and mast cells is frequently observed in humans [29,30] and experimental colitis, including murine *Trichinella spiralis*-induced colitis [27]. Following TNBS colitis, eosinophils and T cells are commonly found adjacent to myenteric ganglia. This indicates a specific targeting to enteric ganglia [31]. Eosinophils are first observed adjacent to myenteric ganglia at six hours, and T cells are observed at twenty-four hours. There are no eosinophils associated with myenteric ganglia in normal intestine, and T cells are rarely found in the vicinity. Thus, their presence in elevated numbers is an indication of ganglionitis and suggestive of neuropathology. In addition to ganglia, immune cells have also been observed in smooth muscle layers in colitis [27,32].

The therapeutic efficacy of leukocyte inhibitors has been demonstrated in murine models of colitis. In addition, in patients with IBD, the anti-inflammatory effects of 5-aminosalicylic acid compounds [33] and corticosteroids [34] have been ascribed, at least in part, to their ability to inhibit the formation of and/or scavenging of reactive oxygen metabolites from neutrophils and to reduce neutrophil infiltration, respectively. Interestingly, reducing neutrophil granulocyte infiltration into the intestinal mucosa by pretreatment of mice with anti-neutrophil serum results in partial attenuation of myenteric plexus cell loss following DNBS colitis [35]. Moreover, resolution of the inflammatory process is associated with a gradual improvement in propulsive action.

## Mechanisms of cell death

Inappropriate neuronal cell death occurs in various neurodegenerative and inflammatory conditions of the nervous system. While the mechanisms of neuronal cell loss have not been studied in animal models of colitis or in patients with IBD, investigation of neurodegenerative and inflammatory conditions of the central and peripheral nervous systems have frequently demonstrated activation of apoptotic pathways.

Caspase-3 is a pivotal mediator of apoptosis because of its ability to cleave a vast array of proteins. Active caspase-3 consists of 17 and 11 kDa subunits derived from a 32 kDa pro-enzyme by cleavage at multiple aspartic acid sites. Its cleavage results in the functional loss of multiple proteins, leading to the death of the cell.

Recently, De Giorgio et al. demonstrated neuronal apoptosis in guinea pig myenteric plexus neuron cultures [36]. Furthermore, this myenteric neuronal cell loss was mediated through activation of a caspase-3-dependent pathway. Strongly activated caspase-3 and cleaved poly (ADP-ribose) polymerase immunoreactivities were evident as early as 30 minutes following instillation of dinitrobenzene sulfonic acid (DNBS) to induce experimental colitis. Lourenssen et al. recently demonstrated that the caspase inhibitor zVAD, but not DEVD, significantly prevents neuronal death, implying a largely caspase-3/7 independent mechanism of apoptotic death. This finding was supported by staining for annexin V and cleaved caspase-3 [37].

Transglutaminases are a large family of related and ubiquitous enzymes which catalyze the cross linking of a glutaminyl residue of a protein/peptide substrate to a lysyl residue of a protein/peptide co-substrate [38]. These enzymes are also capable of catalyzing other reactions that are important for cell life. Recently, "tissue" transglutaminase (tTG) was shown to be involved in molecular mechanisms responsible for very widespread human pathology in celiac disease, in addition to a number of human neurodegenerative diseases.

Experimental colitis upregulates tTG and increases its activity in the ENS [23]. One week after TNBS-induction of colitis in rats, myenteric neurons display increased tTG compared to control and non-inflamed colon. Similarly, tTG activity is significantly higher in inflamed colon. In cultured myenteric neurons incubated with retinoic acid, a tTG inducer, significantly increased neuronal apoptosis suggests that tTG enhances neuronal susceptibility to apoptosis. These data pave the way to future therapeutic options targeting neuronal apoptosis as a pathogenic factor that could contribute to neuropathologic changes during gut inflammation.

## Neurochemical and receptor changes

Overall, a considerable body of evidence from animal models and patients with IBD shows that dramatic alterations occur in the ENS in conditions of inflammation, and are coupled with disturbed motility that are likely to play an important role in the pathogenesis of the disease.

Evidence in animal models of IBD shows that, in addition to gross changes in the morphology and architecture of neural ganglia and nerve cell bodies, subtle changes in the expression of neurotransmitters or their receptors at synapses within the gut wall are a prevalent and important aspect of intestinal inflammation. Inflammatory cells in the intestine, such as dendritic cells, lymphocytes, macrophages and mast cells, express receptors for small molecule neurotransmitters and neuropeptides, and enteric neurons are responsive to cytokines that inflammatory cells secrete.

Chemical messengers that have been implicated in IBD include neuropeptides and small molecules such as acetylcholine (ACh) and 5-hydroxytryptamine (5-HT or serotonin) [39]. In the rodent intestine, inflammation markedly affects the cholinergic neurons that comprise the major excitatory phenotype of the ENS, causing decreased release of ACh [40]. This may be derived from changes in the expression of the synaptic vesicle proteins that are necessary for neurotransmitter release, such as the selective decrease of the synaptic vesicle protein neuronal calcium sensory 1 during TNBS-induced colitis [12]. 5-HT, a major gastrointestinal paracrine hormone and enteric neurotransmitter known to be involved in the initiation of peristaltic activity, has recently been recognized to be a pro-inflammatory neurotransmitter as well, and its secretion is increased in the enterochromaffin cells of patients with CD [41].

Substance P (SP), which belongs to a family of structurally linked peptides known as tachykinins, is an 11-amino acid peptide secreted by nerves and inflammatory cells such as monocytes, macrophages, eosinophils, and lymphocytes, and acts by binding to the neurokinin-1 (NK-1) receptor [28]. In addition to a variety of modulatory effects (i.e., smooth muscle contraction, vasodilation, and epithelial ion transport), SP is a mediator of neurogenic inflammation and plays an important role [28] in inflammatory diseases of the respiratory, gastrointestinal, and musculoskeletal systems [28].

Elevated SP and upregulated NK-1 receptor expression have been reported in the rectum and colon of patients with IBD [42]. SP increases dramatically in the myenteric plexus of the colon of patients with UC and involves a shift from a mainly cholinergic to a more extensive SP innervation [43,39]. In fact, the density of SP nerve terminals in the lamina propria of UC patients correlates with the severity of the disease. Interestingly, similar changes in neurochemical coding were also observed in non-inflamed areas of bowel and may constitute part of the neuronal basis for the altered motility disturbance observed during UC [43]. There is also an increased expression of SP binding sites in the inflamed mucosa of UC patients [44] as well as an increase in NK-1 receptor mRNA [19]. As a result of these findings, tachykinin antagonists have been proposed as potential anti-inflammatory compounds [45].

In support of the idea that SP is pro-inflammatory and plays a role in the pathogenesis of IBD, specific antagonists for the high-affinity NK1 receptor decrease inflammation and severity of DSS-induced colitis, protect from dinitrofluorobenzene- (DNFB-) induced colitis in mice [42] and protect SCID mice against T-cell induced chronic colitis [46]. In addition, blocking NK1 receptors also results in reductions in colitis in acetic acid-induced colitis and *Clostridium difficile *ileitis. Neuron-derived pro-inflammatory SP can stimulate macrophages, mast cells, and endothelium to release cytokines, which can contribute to and sustain an inflammatory infiltrate, thereby causing tissue damage and neurodegeneration. Since NK-1 promotes inflammation, NK-1 antagonists are candidates to be therapeutic agents for IBD. However, whether they attenuate the effects of inflammation in the ENS is unknown.

Considerable evidence suggests that vasoactive intestinal peptide (VIP) also participates in the pathophysiology of IBD [47]. VIP is a 28-amino-acid peptide belonging to the pituitary adenylate cyclase-activating polypeptide (PACAP)/glucagon superfamily based on sequence homologies. All layers of the colon contain VIP with the highest concentration in the myenteric plexus. Functionally, VIP inhibits the peristaltic reflex in the circular muscle layer, controls intestinal blood flow, and modulates the immune system by binding to two G-protein-coupled VIP receptor -- VPAC1 and VPAC2 -- which are also shared by PACAP. VIP is released from nerve terminals that contain nitric oxide synthase (NOS). Together, these two peptides are believed to be the primary components of non-adrenergic, non-cholinergic nerve transmission in the gut.

Several pieces of evidence indicate that VIP participates in the pathophysiology of colitis and IBD. Treatment with VIP after the onset of TNBS-induced colitis in mice reduces the clinical and histopathologic severity of colitis as well as Th1 cytokine levels [48]. In addition, glucagon-like peptide 2 (GLP-2), an important regulator of nutritional absorptive capacity with anti-inflammatory actions, reduces intestinal mucosal inflammation in TNBS-induced colitis in rats by activation of VIP neurons of the submucosal plexus [49]. These findings indicate the potential use of VIP in the therapy of CD; however, VIP has potential side effects, including hypotension and diarrhea, when given at high doses.

Significant increases in VIP content of colonic nerves have been reported in biopsies from CD patients compared to UC patients or controls [50]. A similar increase in VIP concentration was also evident in rectal biopsies from CD, but not UC, patients. Similar findings have been reported in the myenteric plexus in guinea-pig TNBS colitis [21]. Kishimoto et al. reported an increased VIP immunoreactivity in neurons and nerve fibers in both plexuses of the colon and an elevated content of VIP in dextran sulfate sodium (DSS)-induced colitis in rats [51]. In contrast, Mazumdar and Das found decreased expression of VIP immunoreactivity in the colon of both CD and UC patients, compared to controls [52], while two other studies showed a significant decreased of rectal and colonic VIP in UC and CD patients.

The mechanism by which inflammation selectively alters the expression of transmitters in the ENS is currently unknown; however, a recent study of TNBS colitis in rats identified a role for nerve growth factor and its receptors in susceptibility to inflammation-induced neuronal damage.

In addition to SP and VIP, mRNA for a number of peptides is increased in colon tissues from mice undergoing experimental colitis induced by oil of mustard (OM; allyl isothiocyanate), a direct stimulant of small nerve fibers and a potent, acute inflammatory irritant. OM has been used experimentally to evoke allodynia and visceral hyperalgesia following intracolonic administration to mice [53]. Intracolonic application of a 0.5% solution of OM produces a severe, transient colitis with the greatest damage within the first three days.

OM colitis has a vital neuronal component, as evidenced by elevated expression of mu- and delta-opioids, and the receptors NK1, cannabinoid receptor 1 (CB1R) and TRPA1, the receptor for OM and a member of the transient receptor potential channel family. TRPA1 is responsible for detecting physical signals such as noxious cold and directly contributes to the cold hyperalgesia present in inflammatory states. Intrathecal injection of TRPA1 antisense oligonucleotides, reduces TRPA1 expression and attenuates visceral hyperalgesia following TNBS-induced colitis [54].

Neuronal receptor and neuropeptide mRNA expression are upregulated within the first two hours following OM colitis [53]. CB1R is transiently increased, consistent with other reports related to pathways associated with pain sensation stimulated by colitis, and also consistent with data demonstrating increased CB1R expression in human IBD.

## Alterations in enteric neural signaling

A number of electrophysiological studies have been performed in animal models of IBD in order to elucidate the mechanisms underlying inflammation-induced changes in gut motility. Independent of the method used to induce colitis, the type of enteric neuron most dramatically affected by inflammation is the afterhyperpolarizing (AH) neuron.

In the normal guinea pig intestine, the AH neuron, named for its prolonged afterhyperpolarization, functions as an intrinsic primary afferent neuron in the myenteric plexus and is associated with peristalsis, mucosal secretion, and vasodilation. These neurons very rarely receive fast synaptic inputs in normal conditions, but in the guinea pig distal colon more AH neurons receive fast synaptic inputs following the induction of inflammation [55]. Similar findings have been reported for a *T. spiralis *model of jejunitis. In this model, AH myenteric neurons exhibit increased excitability, depolarized membrane potential, and reduced AH potential amplitude and duration along with increased input resistance [56]. AH neurons in the inflamed ileum also exhibit another form of prolonged excitation, a prolonged hyperexcitability after a brief stimulus that lasts up to three hours [57].

AH neurons characteristically receive slow excitatory postsynaptic potentials (EPSPs) in the normal bowel. However, maintained excitation that lasts from 27 minutes up to three hours occurs in AH neurons in inflamed bowel [57]. Such long-term hyperexcitability is not encountered in enteric neurons of normal intestine. The maintained excitation indicates that a brief synaptic activation can trigger a long period of hyperexcitability in AH neurons after they have been exposed to an inflamed environment. These findings suggest that perturbation of the sensory component of intrinsic motor reflexes may occur during inflammation. Maintained neuronal excitation, observed only in neurons from the inflamed bowel, may contribute to the dysmotility, pain, and discomfort associated with intestinal inflammation and IBD in particular. The mechanisms responsible for the changes in excitability are not yet understood, but they may involve a persistent alteration in channel expression and/or a continuous release of inflammatory mediators due to low-grade inflammation.

## Alterations in sympathetic neural activity

IBD alters the function of the ENS and the sensory innervation of the GI tract. Less is known about whether IBD affects the sympathetic nervous system, although experimental evidence of sympathetic neural dysfunction in IBD is increasing [58]. A decrease in the release of noradrenaline from sympathetic varicosities in inflamed and un-inflamed regions of the GI tract has consistently been reported for animal models of colitis. Recent findings suggest that the decrease in neurotransmitter release may be due to inhibition of N-type voltage-gated Ca2 c + current in postganglionic sympathetic neurons [59]. How an alteration of sympathetic function contributes to the pathogenesis of IBD has not yet been determined.

## Neuroimmune factors

It has been known for some time that the ENS and mucosal immune systems have the ability to regulate one another's functions. Nerve cells are found in close proximity to immune cells in the mucosa. The two systems even share several chemical mediators, such as SP. Neuronal activation can lead to degranulation of mast cells and influx of neutrophils, thereby recruiting elements of innate immunity to the area. Lymphocytes express receptors for neuropeptides released by enteric nerves, and stimulation of these cells with SP or VIP can induce their differentiation and alter their production of immunoglobulins.

Signaling between immune cells and enteric neurons can also evoke alterations in gut function. Linden et al. indicated that the hyperexcitability of intrinsic primary afferent neurons of inflamed guinea pig colon may be secondary to activation of cyclooxygenase (COX)-2 and production of prostaglandins (PGE_2_) [60]. Alterations in electrical and synaptic properties of enteric neurons in non-inflamed colon of guinea pigs with TNBS-induced ileitis are accompanied by significantly increased PGE_2 _tissue levels, despite the lack of overt inflammation in the colon [61]. Moreover, these increased PGE_2 _levels are attenuated in the presence of COX2 inhibitors.

Ileitis also increases the number of colonic 5-HT-immunoreactive enteroendocrine cells and release of 5-HT, despite the absence of inflammation in the colon. Therefore, increased prostaglandin and 5-HT levels may underlie some of the changes in neuronal properties observed at sites of gut inflammation. These changes can occur in non-involved regions during episodes of intestinal inflammation.

Several studies point to the involvement of kinases in the sustained changes that occur following inflammation [62]. In previously inflamed colon, PKA activity in nerve terminals increases and contributes to the facilitation of fast synaptic transmission, possibly through inhibition of big conductance K^+ ^channels and an increase in the release-ready pool of synaptic vesicles. In other studies, inflammation of the small intestine induced by *Trichinella spiralis *causes increased adenylyl cyclase activity of myenteric neurons 6-9 days after infection [63].

### Mast cells

Mast cells and their chemicals have the potential to mediate the effects of inflammation on enteric nerves because they function as intermediaries between neurons and the inflammatory soup in their environment [64]. Both mast cells and neurons can be increased or decreased by an inflammatory environment and, upon activation, release mediators that can act on the gut neuromuscular apparatus. Although mast cells are most widely known for their role in allergic responses, these cells are normally present throughout the gut and are involved in a range of physiological and pathological activities including mucosal defense mechanisms and inflammation.

Enteric mast cells are concentrated with granules that serve as sites of storage for a wide mix of preformed chemical mediators. Antigens stimulate the mast cells to release mediators, which then diffuse into the extracellular space to influence other cell types. Mast cells may release an array of inflammatory mediators, which may stimulate the residential macrophages on the one hand and intrinsic and extrinsic neurons, on the other hand, which may ultimately result in GI dysfunction and symptoms.

Mast cell degranulation evoked by psychological stress activates an "alarm program" in the ENS to produce symptoms of diarrhea and abdominal distress. Mast cells are located close to enteric nerves and provide a structural basis for communication between the gut and the ENS. They release mediators, which signal the ENS that degranulation has taken place while simultaneously attracting immune/inflammatory cells into the intestinal wall from the mesenteric circulation. Blockade of ENS and sensory afferents by exposure of gut to the nerve blocker tetrodotoxin, or application of mast cell stabilizing drugs, prevents the acute inflammatory response to *Clostridium difficile *toxin-A. The terminals of vagal and spinal afferent neurons also express receptors for mast cell mediators.

There is considerable clinical evidence for mast cell involvement in human IBD. In the colorectal mucosa from patients with CD and UC, the amount of mast cell tryptase is significantly increased as is the number of mast cells in the lamina propria and submucosa [65]. Increased numbers of mast cells found in the colonic mucosa of IBD patients are accompanied by dramatically increased expression of TNF-alpha, IL-16, and SP [66]. Evidence of mast cell degranulation is found in the intestinal wall of IBD patients, suggesting that mast cell degranulation is involved in the pathogenesis of IBD [66].

Infections with nematode parasites stimulate proliferation of mast cells in animals, which serve as experimental models for the investigation of mast cell functions in detection of and signaling the presence of sensitizing antigens and infectious invaders that broach the mucosal barrier.

Mast cells mediators include a number of proinflammatory substances (tryptase, histamine, platelet activating factor, prostaglandins, leukotrienes) and have the capacity to produce a variety of cytokines. Mast cells undergo degranulation during intestinal manipulation and may be part of the mechanisms responsible for triggering cellular infiltration and subsequent altered bowel motility.

Evidence for communication between mast cells and the ENS is derived from electrophysiological recordings in enteric neurons in intestinal preparations from antigen-sensitized animal models and recordings of the actions of mast cell mediators on the electrical and synaptic activity of ENS neurons. Several mast cell-derived mediators have neuropharmacological actions on the electrical and synaptic behavior of neurons in the ENS including histamine, interleukin-6, leukotrienes, 5-HT, platelet activating factor, mast cell proteases, adenosine, interleukin-1β, and prostaglandins [67]. The evidence suggests that mast cell signals trigger a neural program for defensive intestinal behavior in response to circumstances within the lumen that are threatening the functional integrity of the whole animal. Immunoneural integration progresses sequentially beginning with immune detection followed by signal transfer to the ENS, and then by the selection of a specific neural program for coordinated mucosal secretion and powerful propulsion that effectively clears the antigenic threat from the intestinal lumen [67].

### Enterochromaffin cells

Studies show that intestinal inflammation is accompanied by alterations in enteroendocrine cells. The 5-HT-containing enterochromaffin (EC) cells are the most abundant enteroendocrine cells present in the gut. They are distributed throughout the GI tract, although the greatest concentrations are located in the small intestine and rectum. Ninety-five percent of GI tract 5-HT is found in EC cells with the remainder within enteric neurons.

Enterochromaffin cells are interposed between gut epithelial cells, where they act as sensors of the intraluminal milieu. 5-HT release from EC cells is mediated by luminal or neuronal stimuli that include stimuli such as mucosal stroking, and endogenous chemical stimuli such as adenosine. Following its release, 5-HT activates a variety of receptors and participates in reflex propagation [68]. Enterochromaffin cells are influenced by GI inflammatory conditions. Moreover, changes in the content, release, and re-uptake of 5-HT have been reported in IBD [69].

Mucosal EC cell number is increased in clinical and experimental models of IBD [70]. Enterochromaffin cell number is increased both in affected and non-affected sites of the gut. A further factor that may act to increase 5-HT levels is that expression of the serotonin reuptake transporter (SERT) is reduced.

The SERT molecule is a member of the Na^+^/Cl^- ^neurotransmitter transporter family and is expressed by epithelial cells and neurons in the gut [68]. 5-HT is removed from the interstitium by reuptake via SERT. These changes are important due to the strategic location of EC cells in the gut mucosa, making it likely that 5-HT has a direct action on enteric neurons, regulating gut motility and secretion [70]. Recently, Bischoff et al [71] demonstrated that deletion of SERT increases the severity of TNBS colitis in mice. These data suggest that 5-HT signaling and its SERT-mediated termination may be involved in the pathophysiology of IBD. There is evidence that SERT expression is decreased in human IBD [41] as well as in mice as a result of TNBS-induced colitis [71].

5-HT can modify gut motility/sensation is several ways. 5-HT is also present within enteric nerves [72]. It acts upon 5-HT3 receptors of vagal afferent nerve fibers, which in turn stimulate intestinal secretion and motor reflexes. Furthermore, 5-HT can act on 5-HT3, 5-HT4, and 5-HT1P receptors on enteric neurons thereby contributing to peristalsis and stimulating intestinal transit [73]. These findings implicate 5-HT signaling in the pathophysiology of IBD and suggest that drugs targeting gut 5-HT receptors could benefit patients suffering from inflammation-related gut disorders.

### Other inflammatory cells in IBD

During an inflammatory flare, lymphocytes and other inflammatory cells infiltrate the bowel wall, and both local and circulating cytokine levels (e.g., Il1β, TNF-α, IL-6) are elevated in patients with IBD. These cytokines, secreted by circulating and resident inflammatory cells, may exert direct influences on enteric nerves and are capable of modulating its neuromuscular function.

In addition to increases in mast cells and EC cells, there are trends for increases in CD3^+ ^lymphocytes and the proportion of lymphoid tissue. CD3 is a "pan T-lymphocyte marker" and allows one to estimate the overall number of T-lymphocytes in the lamina propria of patients with IBD. It is possible that specific subclasses of lymphocytes may be altered in IBD (e.g., CD4^+ ^T cells). Recently, circulating and tissue B cells from CD patients were shown to demonstrate elevated basal levels of activation [74].

## Post-inflammatory changes in gut function

There is no question that GI inflammation leads to significant changes in neuron-controlled gut functions, and that alterations in gut function are accompanied by changes in neurophysiological, neurochemical, and morphological properties of enteric nerves. It has recently become apparent that inflammatory changes persist following resolution of the initiating inflammatory event, suggesting that prior inflammation can alter GI function and visceral sensation.

*In vivo*, colitis causes impaired neurotransmitter function in both inflamed and noninflamed regions of the bowel [75], and altered neuronal signaling can persist up to eight weeks after the initiation of colitis, long after inflammation is apparently resolved, according to histological examination and assay of myeloperoxidase activity in tissue extracts [76]. Other animal models also show that neural function can remain affected by prior damage for extended periods of time, such as the impaired ACh metabolism seen at least six months after *Trichinella spiralis*-induced intestinal inflammation [77]. This implies that the inflammation may cause a prolonged change in the intrinsic properties of the neurons. What mediates the persistent post-inflammatory hyperexcitability of enteric neurons is not yet known. However, increased numbers of immune cells in the human rectal mucosa have been documented for at least a year after an acute infection with *Campylobacter enteritis *[78].

A surge of eosinophils and T-lymphocytes associated with the enteric ganglia occurs at 1-7 days following TNBS colitis [31]. However, elevated immune cell numbers occur in the lamina propria of the mucosa until 56 days. Thus, ongoing mucosal inflammatory reaction may contribute to the persistence of enteric neuropathy. This makes sense, since the major neuron type in myenteric ganglia whose properties are changed by a brief severe inflammation are the AH neurons [55]. These neurons have processes in the mucosa that are stimulated by local mediators, for example 5-HT. The exposure of their nerve terminal in the mucosa to the products of the immune cells may contribute to the maintenance of increased excitability of AH neurons. Questions remain as to what genes are being activated or inactivated, and as to how these genes might be influenced to return to normal levels of activity.

## Oxidative stress

It is generally hypothesized that IBD is caused by GI tract immune dysregulation, because the disease is accompanied by a considerable infiltration of inflammatory cells in the gut mucosa [79]. However, the specific pathways leading to cellular damage have yet to be fathomed. Oxidative stress is a potential biological and/or triggering factor for IBD, because the detrimental effects of reactive oxygen species (ROS) are well established in the inflammation process.

Oxidative stress arises when there is a marked imbalance between the production of ROS and their removal by antioxidants. In reaction to mild oxidative stress, tissues often respond by producing more antioxidants; however, severe persistent oxidative stress depletes body antioxidant resources and overtakes its ability to produce more antioxidants, leading to lower antioxidant levels and injury in the tissues.

For example, patients with IBD demonstrate decreased expression of the anti-oxidant heme-oxygenase-1 (HO-1) in the intestinal epithelium of inflamed colonic mucosa, compared to control specimens, suggesting dysregulated expression in inflammation [80]. HO-1 is the rate-limiting enzyme in the catabolism of heme, which leads to the generation of biliverdin, iron, and carbon monoxide and has been shown to have important anti-inflammatory properties. Moreover, induction of HO-1 protects neurons from oxidative stress-induced cell death, possibly in response to activation of the transcription factor Nrf2 [81].

*In vivo*, induction of HO-1 by the HO-1 inducer cobalt protoporphyrin (CoPP) leads to significant down-regulation of colonic inflammation in acute DSS-induced colitis [80]. Moreover, the protective effects of HO-1 in experimental colitis are replicated by administration of hemin, which markedly reduces programmed cell death of colonic epithelium and attenuates the production of interleukin 17 [82]. In addition, rectal administration of tranilast, a mast cell stabilizer, significantly increases expression of HO-1 in colonic epithelial cells and is thought to mediate the anti-inflammatory effects of tranilast in DSS colitis and human IBD [83].

**Figure 1:**
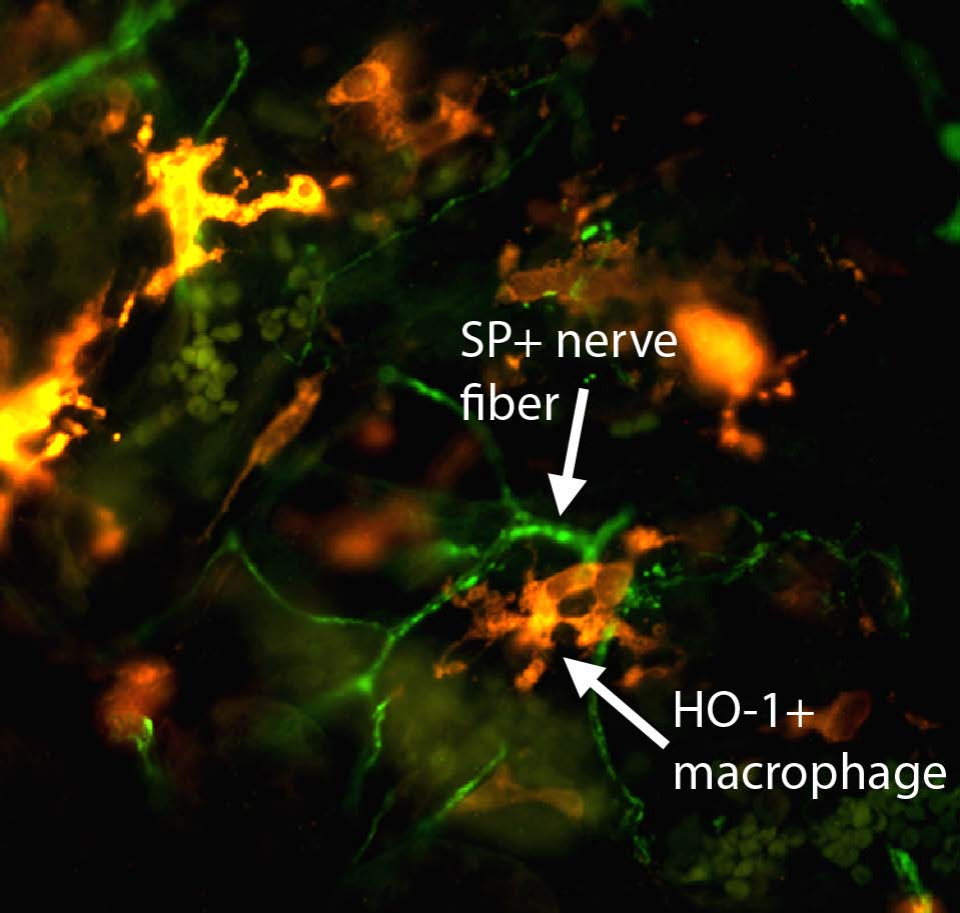
**Immunoreactivity to hemeoxygenase-1 (HO-1) and substance P (SP) in a cryostat section of human colon**. An HO-1-positive macrophage (orange) is seen in close proximity to a SP-containing nerve fiber (green) in the colonic mucosa.

In addition to colonic epithelial cells, HO-1 is expressed in macrophages. In both mouse and human colon, HO-1 immunoreactivity is displayed by macrophages in close proximity to SP-containing nerve fibers (Figure 1). Macrophages have pro- and anti-inflammatory activities, depending on their phenotype. For example, CD206^+^/HO-1^+ ^gastric macrophages protect against oxidative stress-induced damage and are required for prevention of diabetic gastroparesis in mice [84]. Thus, expression of HO-1 in macrophages could constitute an important component of the anti-inflammatory effect by increasing antioxidant protection and decreasing the inflammatory component of IBD lesions. Moreover, the expression of HO-1 in macrophages close to enteric nerves could act as a natural defense mechanism to alleviate enteric nerve injury in the GI tract. Therefore, induction of HO-1 expression in macrophages might be a therapeutic option to protect neurons in patients with IBD; however, this idea remains to be tested.

The presence of reactive oxygen metabolites, or the molecules damaged by them, has been extensively studied in patients with IBD [85]. Although some results are conflicting, it seems that patients with IBD demonstrate excessive oxidized molecules compared with healthy control subjects in a variety of organ systems. This seems to be more pronounced in patients with CD. In fact, the most commonly used drug for patients with IBD, 5-aminosalicylic acid, has ROS scavenging capabilities [86].

Similar results have been found in animal models of IBD, which universally show abnormal levels of antioxidants or oxidized molecules [85]. The most striking evidence comes from genetic knockout mice lacking the antioxidant enzyme of glutathione peroxidase. These animals display destructive colitis similar to UC as early as 11 days of age [87].

Activation of immune cells and the release of ROS such as H_2_O_2 _are prominent early events in the pathogenesis of IBD that could affect the ENS. There is a close parallel between the loss of enteric neurons and their axons after exposure to H_2_O_2 _observed *in vitro*, and the damage to the ENS that is seen in the acute phase of initiation of colitis *in vivo*, where a significant loss of neurons occurs by 24 h [24]. The damage produced by H_2_O_2 _does not target a specific neuronal phenotype as the death of myenteric neurons is distributed equally among cholinergic and nitrergic neurons. Thus, the neurotoxic events of inflammation appear to be nonselective.

A comparison of ROS levels, using DHR fluorescence, in preparations of myenteric plexus from 12- to 15-month-old rats showed increased levels of free radical generation with age, suggesting that aging correlates with higher levels of intrinsic neuronal ROS. Interestingly, elevated levels of intraneuronal ROS are correlated with substantial neuron loss suggesting a plausible mechanism whereby age-related neuron cell death is produced [88].

Enhanced ROS production and oxidative injury play a cardinal role in the onset and progression of neurodegenerative disorders. To maintain a proper redox balance, the CNS is equipped with an antioxidant defense mechanism consisting of endogenous antioxidant enzymes. The expression of most antioxidant enzymes is tightly controlled by the antioxidant response element (ARE) and is activated by Nrf2, a transcription factor that regulates an expansive set of antioxidant genes that act in synergy to remove ROS through sequential enzymatic reactions. When activated, Nrf2 specifically targets genes bearing an ARE within their promoters such as HO-1, 1-ferritin, and glutathione peroxidase, which maintain redox homeostasis and influence the inflammatory response. Not surprisingly, Nrf2-deficient mice have an increased susceptibility to DSS-induced colitis, possibly due to reduced expression of antioxidant phase II detoxifying enzymes with a concomitant increased induction of proinflammatory mediators [89]

Several of the genes commonly regulated by Nrf2 have been implicated in protection from neurodegenerative conditions. For this reason, Nrf2 may be considered a therapeutic target for conditions that are known to involve free radical damage. Mitochondrial dysfunction and build-up of ROS are indicative of neurodegeneration; therefore, targeting Nrf2 may be valuable in combating neurodegeneration in IBD.

## Neuroprotection by enteric glial cells

Enteric glial cells have recently been postulated to have a novel neuroprotective role in the ENS. Enteric glial cells are analogous to, and share many similarities with, astrocytes of the CNS. Following major ablation of enteric glial cells, there is a significant decrease in the number of myenteric neurons compared to control [90]. Notably, there is a loss of NOS-containing neurons in the myenteric plexus, which likely underlies disturbances observed in smooth muscle relaxation and intestinal transit time. The mechanisms responsible for neuronal cell loss remain unknown. However, this may be due to reduced availability of neuroprotective factors, which could lead to an increase in susceptibility of enteric neurons to insults such as oxidative stress, as occurs in IBD.

Enteric glial cells are known to actively participate in inflammatory processes. They produce and respond to an array of cytokines. They also protect neurons from oxidative stress in part via reduced glutathione (GSH) [91]. GSH has been identified in the CNS as being synthesized by astrocytes and exerting neuroprotective effects, especially during oxidative stress [92].

## Conclusion

This review describes changes in the anatomical and physiological properties of enteric neurons in response to gut inflammation. In general, inflammation-related changes in GI function likely involve neurodegeneration and neuroplasticity in the ENS, as well as changes in the structure and function of enteric glia. Although the mechanisms involved in modulation of enteric neural activity during inflammation are not completely understood, oxidative stress appears to play an important role in the process. Thus, it is possible that neuroprotective agents that curtail oxidative stress in the ENS could restore gut function and could have utility in the treatment of gut dysfunction in IBD.

## Abbreviations

5-HT: 5-hydroxytryptamine or serotonin; ACh: acetylcholine; AH: afterhyperpolarizing; ARE: antioxidant response element; CB1R: cannabinoid 1 receptor; COX-2: cyclooxygenase-2; DNBS: dinitrobenzene sulfonic acid; DSS: dextran sulfate sodium; DRG: dorsal root ganglia; EC: enterochromaffin; ENS: enteric nervous system; EPSP: excitatory postsynaptic potential; GI: gastrointestinal; GSH: glutathione; HO-1: hemeoxygenase-1; IBD: inflammatory bowel disease; NK-1: neurokinin-1; NK1R: neurokinin 1 receptor; Nrf2: nuclear factor erythroid-related factor 2; OM: oil of mustard; PACAP: pituitary adenylate cyclase-activating polypeptid; PGE_2_, prostaglandin; SP: substance P; TNBS: trinitrobenzene sulfonic acid; ROS: reactive oxygen species; tTG: tissue transglutaminase; VIP: vasoactive intestinal peptide.

## Competing interests

The authors declare that they have no competing interests.

## Authors' contributions

SEL and AK participated in the preparation of the manuscript. All authors read and approved the final manuscript.
